# LucKi Birth Cohort Study: rationale and design

**DOI:** 10.1186/s12889-015-2255-7

**Published:** 2015-09-21

**Authors:** Dianne de Korte-de Boer, Monique Mommers, Huub MH Creemers, Edward Dompeling, Frans JM Feron, Cindy ML Gielkens-Sijstermans, Mariëlle Jaminon, Suhreta Mujakovic, Onno CP van Schayck, Carel Thijs, Maria Jansen

**Affiliations:** Department of Epidemiology, CAPHRI School for Public Health and Primary Care, Maastricht University, PO Box 616, 6200 MD Maastricht, The Netherlands; Department of Youth Health Care, South Limburg Public Health Service, Geleen, The Netherlands; Department of Paediatric Respiratory Medicine, CAPHRI School for Public Health and Primary Care, Maastricht University Medical Centre, Maastricht, The Netherlands; Department of Social Medicine, CAPHRI School for Public Health and Primary Care, Maastricht University, Maastricht, The Netherlands; Department of Environmental Health, South Limburg Public Health Service, Geleen, The Netherlands; Orbis Child and Youth Health Care, Orbis Medical Concern, Sittard, The Netherlands; Department of Research and Development, South Limburg Public Health Service, Geleen, The Netherlands; Department of General Practice, CAPHRI School for Public Health and Primary Care, Maastricht University, Maastricht, The Netherlands; Department of Health Services Research, CAPHRI School for Public Health and Primary Care, Maastricht University, Maastricht, The Netherlands

**Keywords:** Study protocol, Birth Cohort, Asthma, Childhood, Atopic diseases, Overweight, Public health

## Abstract

**Background:**

Infancy and childhood are characterized by rapid growth and development, which largely determine health status and well-being across the lifespan. Identification of modifiable risk factors and prognostic factors in critical periods of life will contribute to the development of effective prevention and intervention strategies.

The LucKi Birth Cohort Study was designed and started in 2006 to follow children from birth into adulthood on a wide range of determinants, disorders, and diseases. During preschool and school years, the primary focus is on the etiology and prognosis of atopic diseases (eczema, asthma, and hay fever) and overweight/obesity.

**Methods/Design:**

LucKi is an ongoing, dynamic, prospective birth cohort study, embedded in the Child and Youth Health Care (CYHC) practice of the ‘Westelijke Mijnstreek’ (a region in the southeast of the Netherlands). Recruitment (1–2 weeks after birth) and follow-up (until 19 years) coincide with routine CYHC contact moments, during which the child’s physical and psychosocial development is closely monitored, and anthropometrics are measured repeatedly in a standardised way. Information gathered through CYHC is complemented with repeated parental questionnaires, and information from existing registries of pharmacy, hospital and/or general practice. Since the start already more than 5,000 children were included in LucKi shortly after birth, reaching an average participation rate of ~65 %.

**Discussion:**

The LucKi Birth Cohort Study provides a framework in which children are followed from birth into adulthood. Embedding LucKi in CYHC simplifies implementation, leads to low maintenance costs and high participation rates, and facilitates direct implementation of study results into CYHC practice. Furthermore, LucKi provides opportunities to initiate new (experimental) studies and/or to establish biobanking in (part of) the cohort, and contributes relevant information on determinants and health outcomes to policy and decision makers. Cohort details can be found on www.birthcohorts.net.

## Background

Infancy and childhood are characterised by rapid growth and development, and are considered critical developmental periods in life that strongly contribute to health status, well-being, and behaviour across the lifespan [1]. In fact, many common diseases and challenges in adult life can be traced back to early childhood [2]. Because growth and development in early life are highly influenced by the child’s environment, identification of modifiable risk factors (e.g. in lifestyle, and physical and social environment) forms the basis for the development of preventive measures for childhood and adult diseases.

The LucKi Birth Cohort Study was designed and started in 2006 to follow children from birth into adulthood. Within LucKi, information on a wide range of determinants and outcomes is gathered in order to answer etiological questions and to identify prognostic factors and modifiable risk factors for various childhood and adult diseases and conditions. In time, the LucKi cohort will be large enough to enable new (intervention) studies within part of the study population. Ultimately, the LucKi database will contain relevant information on childhood and adulthood well-being, health and disease that can help researchers, clinicians, and policy makers to develop and implement prevention and intervention measures.

In preschool and early school age, the primary focus of LucKi is on atopic diseases (eczema, asthma, and hay fever) and overweight/obesity. Atopic diseases are among the most prevalent chronic disorders in childhood and their prevalence is still increasing in many developed countries [3, 4]. While some children outgrow their complaints with increasing age, others persist to suffer from atopic diseases into adulthood [5]. Also, childhood overweight and obesity are becoming increasingly prevalent chronic disorders in developed societies [6], and increase the risk of long-term adverse conditions, including cardiovascular, metabolic, pulmonary, and gastrointestinal diseases [7]. It is still largely unknown what factors are involved in the aetiology and/or are responsible for the progression of atopic diseases, and there is even less knowledge on how atopic diseases and overweight/obesity can be prevented or treated. This knowledge is essential for developing effective prevention and intervention strategies and testing these in a real-life setting. Therefore, within LucKi, the main objectives are to 1) to estimate the contribution and timing of known risk factors, and 2) to identify and evaluate new risk factors for atopic diseases and overweight/obesity. The latter requires add-on modules (e.g. questionnaires, measurements, biosampling) to the existing LucKi infrastructure that specifically target potential new risk factors.

Suffering from atopic diseases, overweight or obesity, or other adverse conditions in childhood, as well as acquiring certain lifestyle patterns early in life, may have lifelong consequences. Therefore, in older children and adolescents also other outcomes will be studied, for example social functioning, and behavioural and mental health conditions.

This paper describes the design of the LucKi Birth Cohort study, presents baseline characteristics and prevalence of atopic diseases and overweight in part of the study population, and discusses strengths and limitations of the study.

## Methods/Design

### Study design

LucKi is an ongoing, dynamic, prospective birth cohort study. Since the start in 2006, newborns are continuously being included into the study and will be followed prospectively until at least the age of 19 years. Given the ongoing character of the study, no end date or maximum number of inclusions has been set. Cohort details can also be found on www.birthcohorts.net.

### Setting

**Fig. 1:**
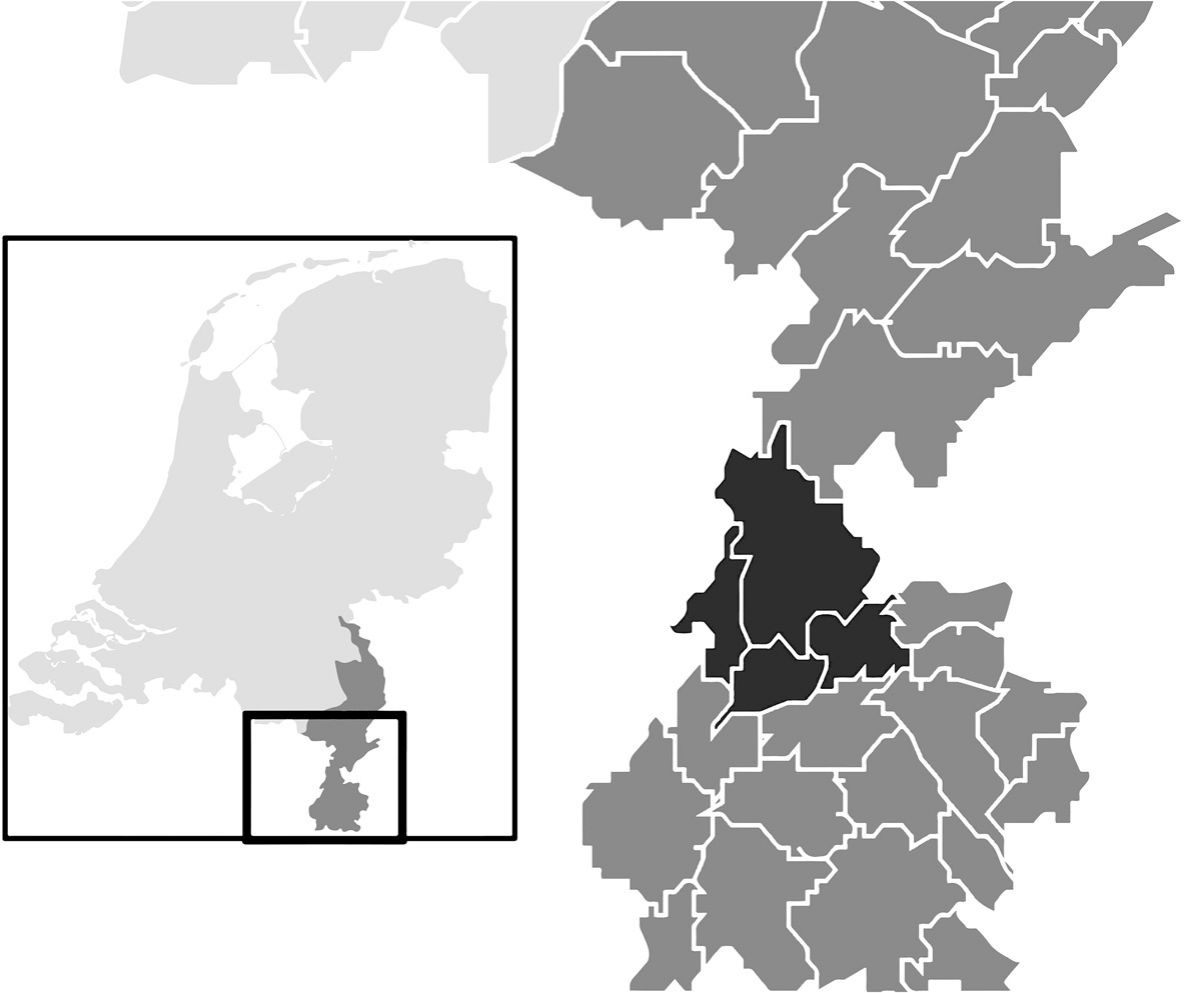
Location of the study area ‘Westelijke Mijnstreek’ in the Netherlands

LucKi is embedded in the Child and Youth Health Care practice of the Westelijke Mijnstreek in the Netherlands. The Westelijke Mijnstreek (Fig. 1) is a region in the Province of Limburg in the south east of the Netherlands that encompasses four municipalities: Sittard-Geleen, Stein, Beek, and Schinnen, with a total area of ~150 km^2^ (~58 square miles). This former coal-mining region is now characterized by a high building density, a relatively dense transport infrastructure, and car and chemical industry. These characteristics, further strengthened by adjacent foreign industrial areas, result in relatively high concentrations of air pollutants, such as nitric oxide and nitrogen dioxide [8].

Due to ageing of the population, the number of inhabitants has been gradually decreasing since the late 1990s. In 2006 the total population size of the Westelijke Mijnstreek was ~154,000; in 2013 there were ~149,000 inhabitants. The large majority (97 %) of inhabitants of the Westelijke Mijnstreek are of Dutch nationality, but 19 % of inhabitants are first or second generation immigrants. Although these figures are comparable to national average (96 and 21 %, respectively), in the Westelijke Mijnstreek more immigrants originate from western countries (75 %) than from non-western countries (25 %), while in the whole of the Netherlands this ratio is 45 versus 55 %. Furthermore, compared to the national average, inhabitants of the Westelijke Mijnstreek have a slightly lower educational and income level, but unemployment rates are comparable. Table 1 illustrates that the prevalence of certain health and life style characteristics, such as overweight and obesity and respiratory diseases is relatively high in this part of the Netherlands [9].

**Table 1:** Health and lifestyle characteristics of inhabitants (between 19 and 65 years of age) of the Westelijke Mijnstreek and the whole of the Netherlands

	Westelijke Mijnstreek	the Netherlands
Perceived health: good/very good	77.7 %	80.7 %
High blood pressure	14.5 %	12.6 %
Chronic respiratory complaints	9.5 %	7.8 %
Diabetes Mellitus	4.6 %	3.6 %
Never drinks alcohol	15.8 %	16.5 %
Heavy alcohol use	12.7 %	11.6 %
Smoker	25.5 %	25.5 %
Heavy smoker	5.5 %	4.7 %
Overweight (incl. obese)	48.5 %	45.5 %
Obese	13.3 %	11.8 %

Data over the year 2012, standardised for age and sex. Data obtained from regional Public Health Services (GGD), Statistics Netherlands (CBS), and the National Institute for Public Health and the Environment (RIVM)

#### Dutch child and youth health care system

Child and Youth Health Care (CYHC) in the Netherlands is obliged by law to promote and protect the health and physical, cognitive and psychosocial development of children between 0 and 19 years of age, and to carry out the national immunisation program. To achieve these goals, children are followed prospectively from birth.

All children living in the Netherlands and their parents or caregivers are invited regularly for a routine physical and developmental check-up. A team consisting of physicians, nurses, and assistants routinely check the child’s growth and development, and provide parents with requested and unrequested support and advice. The first contact with CYHC (when the child is 1–2 weeks old) takes place at home; all other contact moments take place at CYHC-centres. The frequency of routine contact moments decreases with age (see Table 2), but if necessary, children are invited more often to visit CYHC, e.g. when growth is impaired or there are other concerns. In case of suspicion of diseases or disorders, the child is referred to a general practitioner or medical specialist. In general, ~90 % of all parents visit CYHC with their children regularly. The last planned visit takes place when the child is 14 or 15 years old. Children between 16 and 19 years are only invited for an extra check-up visit if their physical or social development raises concerns.

**Table 2:** Number of planned contact moments with Child and Youth Health Care (CYCH), number of LucKi questionnaires, and main (research) topics in different age periods

	0-1	1-4	4-12	12-19
	years	years	years	years
Number of routine contact moments CYHC	9	5	3	2
Number of LucKi questionnaires	2	2	2	1
Main topics CYHC				
General physical examination	++	++	++	++
Growth^a^	++	++	++	++
Overweight & obesity	+	+	+	+
Pre-/peri-/postnatal complications	++			
Family history of diseases/disorders	++			
(Mal) nutrition	++	++	+	+
Vision & hearing	+	+	+	+
Motor development^b^	+	++	+	+
Language development		+	++	
Social class & family functioning	++	++	++	+
Emotional & social development	+	++	++	+
Learning disabilities			++	+
Behavioural problems		+	+	+
Child abuse & neglect	++	++	++	+
Addiction			+	++
Main topics LucKi questionnaires				
Parental medical history	+			
Circumstances around pregnancy & birth	++			
Parental lifestyle characteristics	++	+		
Eczema, wheeze/asthma, hay fever^c^	++	++	++	++
Infections, diarrhoea, fever	++	++	+	+
Other diseases	+	+	++	++
Medication use	+	+	+	+
Diet	++	++	++	++
Physical activity		+	+	+
Day care attendance	+	+		
Indoor environment	+	+	+	+
Outdoor environment	+		+	
School absence			+	+

++ high level of interest; + routine level of interest

^a^ Standardised height, weight, and head or waist circumference measurements by trained personnel at each visit

^b^ Van Wiechen classification of psychomotor development [13]

^c^ ISAAC (the International Study of Asthma and Allergies in Childhood) core questions [10]

All relevant information is kept in a personal digital file in the CYHC registry. After the child reaches the age of 19 years, the file is no longer updated, but is kept for another 15 years in the registry.

### Study population

The study population of the LucKi Birth Cohort study consists of all children born since July 2006 who live in the study area and whose parents agree to participate.

#### Recruitment

Recruitment of the newborns and their parents takes place during the routine home visit by a CYHC-nurse, when the baby is 1–2 weeks old. Purposes of this home visit include to get acquainted with the parents and introduce them to CYHC procedures, and to perform the neonatal heel prick. CYHC receives a notification of an infants’ birth in their region as soon as the parents have registered the newborn child at the municipal office. This registration is obligatory in the Netherlands and has to take place within 3 days after birth. Upon notification CYHC contacts the parents to plan a home visit. Parents receive oral and written information about the purpose and methods of the study and are invited to participate. If parents agree to participate, they are asked to sign informed consent for the use of data from the CYHC registry, pharmacy, hospital and/or general practice.

Parents of children who are not included/invited to participate at birth, but visit CYHC from a later age onwards (e.g. because they move into the study area), are also invited to participate in LucKi from the moment they first visit CYHC in the study area.

#### In- and exclusion criteria

The only inclusion criterion for participating in LucKi is living in the study area. Children whose parents never visit CYHC are excluded.

### Data collection

Data collection takes place through repeated parental questionnaires and is complemented with information from the CYHC registry, pharmacist, hospital and/or general practitioner, provided informed consent to do so is given by the parents. The timing of the questionnaires coincides with routine contact moments in CYHC.

#### Baseline questionnaire

At baseline (1–2 weeks after birth), parents are asked to complete a questionnaire that includes questions on parental medical history, parental characteristics and lifestyle, indoor and outdoor environment, and circumstances during pregnancy and around birth (Table 2).

#### Follow-up questionnaires

When the child is 6/7 months, 14 months, 3 years, and 5/6 years old, parents are asked to complete a follow-up questionnaire for their child. Further follow-up questionnaires are being planned for ages 10/11 and 14/15 years. Follow-up questionnaires include questions on atopic diseases, infections, lifestyle, diet, physical activity, indoor and outdoor environment, medication use, health care utilisation, and school absence (Table 2). Questions on atopic diseases are based on the validated ISAAC (The International Study of Asthma and Allergies in Childhood) questionnaire [10], a questionnaire that is widely used in (monitor) studies on childhood asthma, allergic rhinitis, and eczema.

#### Child and Youth Health Care (CYHC) registry

During the CYHC home-visit at 1–2 weeks of age an individual digital file is set up with information on parental background (e.g. ethnicity, education, work status, family and medical history) and circumstances during pregnancy and birth (e.g. pre-pregnancy weight, gestational age at birth, mode and place of delivery) derived from midwife’s and/or obstetrician’s reports. This file is complemented with new information after every subsequent visit to CYHC.

At each visit a nurse assistant measures the child’s height and weight in a standardised way. Weight is measured on a digital baby scale (birth until 18 months, in a lying or sitting position without clothes) or a digital flat scale (18 months and older, in a standing position wearing only underpants or diaper). Height is measured using an infantometer (birth until 18 months, in a lying position) or a microtoise (18 months and older, in a standing position). Scales and microtoise are calibrated yearly and after translocation.

Other important information that is inquired during CYHC visits and registered in the digital file includes the child’s nutritional status and physical and psychosocial development (Table 2).

#### Pharmacy, hospital, and general practice registries

If additional information on prescribed medication, doctor’s diagnosis, or treatment for various conditions and diseases is needed to answer a specific research question, the variables of interest will be obtained from pharmacy, hospital or general practice registries, provided parents gave informed consent to do so.

### Ethical clearance

The LucKi Birth Cohort Study was approved by the Medical Ethical Committee of Maastricht University Medical Centre (MEC 09–4–058). LucKi is designed according to the privacy rules that are stipulated in the Dutch ‘Code of Conduct for Health Research’ [11].

### Preliminary cohort description

Inclusion of newborns and follow-up of LucKi participants is still ongoing. Since the start in 2006, already more than 5,000 children were included in LucKi shortly after birth, reaching an average participation rate of ~65 %. Most parents completed one or more of the follow-up questionnaires (> 75 %), and gave informed consent to use date from other registries (> 80 %).

Table 3 provides baseline characteristics of children that were included in LucKi between July 2006 and December 2011, and presents the prevalence of eczema, wheeze and overweight in these children.

**Table 3:** Baseline characteristics of LucKi participants born between July 2006 and December 2011

	*N* = 4.230
Sex (% male)	50.2 %
Gestational age at birth^a^	
• < 37 weeks	5.8 %
• 37–40 weeks	70.7 %
• > 40 weeks	22.9 %
Maternal smoking during pregnancy	10.5 %
Breastfeeding (exclusive or combined)	
• until age 3 months	35.4 %
• until age 6 months	16.9 %
Parental history of atopy^b^	57.0 %
Number of older siblings^a^	
• 0	43.2 %
• 1	38.3 %
• > 1	11.9 %
Day care attendance^c^	55.2 %
Pet keeping^d^	51.4 %
Eczema prevalence^e^	
Age 7 months	14.7 %
Age 14 months	13.3 %
Age 3 years	16.2 %
Wheeze prevalence^e^	
Age 7 months	16.4 %
Age 14 months	18.3 %
Age 3 years	13.0 %
Overweight prevalence^f^	
Age 3 years	7.6 %

^a^ Due to missing values, percentages do not add up to 100 %

^b^ Defined as mother or father ever having asthma, hay fever, or eczema

^c^ Defined as visiting day care weekly at age 7 months of age

^d^ Defined as ≥ 1 pets in the home at baseline (1–2 weeks of age)

^e^ The prevalence of eczema and wheeze is based on parentally reported symptoms through validated ISAAC questionnaires [10]

^f^ The prevalence of overweight was calculated with age- and sex-specific cut-off points for Body Mass Index of a widely used international standard [14]

## Discussion

In this paper we presented the rationale and design of the LucKi Birth Cohort Study. Within LucKi, children are followed prospectively from birth into adulthood through repeated questionnaires and routine registries, with, during preschool and school years, a primary focus on the development of atopic diseases and overweight.

### Strengths and limitations

LucKi is entirely embedded in regular Child and Youth Health Care (CYHC) practice and is linked to other registries, which has certain advantages. First, because recruitment and follow-up coincide with routine contact moments, high participation and follow-up rates are ensured. Second, maintenance costs and time investment are relatively low, permitting continuous inclusion of new participants into LucKi. This results in several (birth year) groups within the cohort, some of which may serve as study population for future experimental studies. Third, the continuous character of the study allows us to adapt or supplement the data collection methods according to new insights in the field or to harmonise with other birth cohort studies. Preferably, LucKi measurements will be extended with the collection of biomaterial in the future. Fourth, because information is gathered through different sources, data entries can be cross-checked for a large number of variables, which strengthens the reliability of the data. And fifth, since CYHC has direct access to a large number of children and at different ages, study results of LucKi may be directly implemented into CYHC practice. Other important strengths of LucKi are the longitudinal design, which allows investigating temporal and causal relationships, and the availability of repeatedly measured weight and height data. The latter eliminates bias associated with self-reporting (e.g. social desirability bias and recall bias), which is a well-known limitation in many population studies on overweight and obesity [12].

A limitation to LucKi (as to any observational study) is the possibility of selection in the study population. Although LucKi reaches relatively high participation rates, a reason for not participating may be not speaking the Dutch language and therefore not being able to complete the questionnaires. Also, parents of children who are hospitalised or under intensive paediatric treatment may not attend the CYHC regularly, and in general parents of higher socio-economic status are more likely to participate in such a study. Descriptive data from the regional Public Health Service will enable comparison of responders and non-responders on several characteristics, the results of which will be reported in future research papers. Although any selection may affect estimated prevalence rates, and therefore also the statistical power and generalizability of study results, it will most likely not bias etiological associations. Furthermore, although CYHC only has access to the children after birth, information on important prenatal factors are covered in the baseline questionnaire.

### Application of study results

The information gathered by the LucKi Birth Cohort Study is valuable for (youth) health care and public health policy. Because children are studied at specific ages that coincide with CYHC contact moments, findings from LucKi that can be translated into parental advice or other preventive measures may directly be incorporated in CYHC protocols and reach a large group of children and their parents at once. Further, study results on (modifiable) risk factors, disease prognosis, and medication use may also be relevant for general practitioners and pharmacists.

Moreover, LucKi’s findings may aid policy and decision makers, who need scientific evidence to develop and implement prevention and intervention strategies. LucKi progressively builds on a database containing policy relevant information on a broad range of determinants and health outcomes that may be beneficial to response to current and future public health issues. Furthermore, LucKi results may contribute to the evidence built up by several international birth cohorts and to the development of guidelines. Collaboration between birth cohorts is especially important to achieve more variation in exposure variables, and in that context, LucKi can contribute with research data from children living in a relatively unfavourable outdoor environment.

### Summary (see also Table 4)

**Table 4:** Summary of the aims of the LucKi Birth Cohort Study

LucKi’s aims in preschool- and early school-age:
1) to study the aetiology and the prognosis of atopic diseases and overweight/obesity
2) to identify modifiable risk factors for atopic diseases and overweight/obesity
Long (er)-term aims of LucKi:
3) to study the long-term consequences of early life exposures and acquired lifestyle patterns
4) to constitute a scientific framework for initialising new (intervention) studies
5) to build a database containing information on childhood and adulthood well-being and diseases that is relevant to researchers, clinicians and policy makers

To summarise, children in the LucKi Birth Cohort Study are prospectively followed from birth in order to gain more insight into risk factors for atopic diseases and overweight development. The aims of LucKi are summarised in Table 4. Because of the ongoing, dynamic character, LucKi can be seen as a scientific framework that provides opportunities to initiate new (experimental) studies and/or to establish biobanking in (parts of) the cohort, and may as well contribute relevant information on determinants and health outcomes to health care professionals, and policy and decision makers.

## Abbreviations

CYHC: Child and Youth Health Care
